# *pilA* Gene Contributes to Virulence, Motility, Biofilm Formation, and Interspecific Competition of Bacteria in *Acidovorax citrulli*

**DOI:** 10.3390/microorganisms11071806

**Published:** 2023-07-14

**Authors:** Yuwen Yang, Nuoya Fei, Weiqin Ji, Pei Qiao, Linlin Yang, Dehua Liu, Wei Guan, Tingchang Zhao

**Affiliations:** 1 State Key Laboratory for Biology of Plant Diseases and Insect Pests, Institute of Plant Protection, Chinese Academy of Agricultural Sciences, Beijing 100193, China; fei_nuoya@126.com (N.F.); jiwqcaas@foxmail.com (W.J.); qp382391128@outlook.com (P.Q.); yanglinlin0330@163.com (L.Y.); liudehua199804@foxmail.com (D.L.); wguan@ippcaas.cn (W.G.); 2 National Nanfan Research Institute (Sanya), Chinese Academy of Agricultural Sciences, Sanya 572024, China; 3 College of Life Sciences, Jilin Normal University, Siping 136000, China; 4 Department of Plant Pathology, Plant Protection College, Shenyang Agricultural University, Shenyang 110866, China; 5 College of Plant Protection, Jilin Agricultural University, Changchun 130118, China

**Keywords:** bacterial fruit blotch, interspecific competition, twitching motility, type VI secretion system

## Abstract

*Acidovorax citrulli*, the causative agent of bacterial fruit blotch, can be divided into two main groups based on factors such as pathogenicity and host species preference. PilA is an important structural and functional component of type IV pili (T4P). Previous studies have found significant differences in *pilA* DNA sequences between group I and group II strains of *A. citrulli*. In this study, we characterized *pilA* in the group I strain pslb65 and the group II strain Aac5. *pilA* mutants, complementation strains, and cross-complementation strains were generated, and their biological phenotypes were analyzed to identify functional differences between *pilA* in the two groups. *pilA* deletion mutants (pslb65*-*Δ*pilA* and Aac5*-*Δ*pilA*) showed significantly reduced pathogenicity compared with the wild-type (WT) strains; pslb65-Δ*pilA* also completely lost twitching motility, whereas Aac5-Δ*pilA* only partially lost motility. In King’s B medium, there were no significant differences in biofilm formation between pslb65-Δ*pilA* and WT pslb65, but Aac5-Δ*pilA* showed significantly reduced biofilm formation compared to WT Aac5. In M9 minimal medium, both mutants showed significantly lower biofilm formation compared to the corresponding WT strains, although biofilm formation was recovered in the complementation strains. The biofilm formation capacity was somewhat recovered in the cross-complementation strains but remained significantly lower than in the WT strains. The interspecies competitive abilities of pslb65-Δ*pilA* and Aac5-Δ*pilA* were significantly lower than in the WT strains; Aac5-Δ*pilA* was more strongly competitive than pslb65-Δ*pilA*, and the complementation strains recovered competitiveness to WT levels. Furthermore, the cross-complementation strains showed stronger competitive abilities than the corresponding WT strains. The relative expression levels of genes related to T4P and the type VI secretion system were then assessed in the *pilA* mutants via quantitative PCR. The results showed significant differences in the relative expression levels of multiple genes in pslb65-Δ*pilA* and Aac5-Δ*pilA* compared to the corresponding WT stains. This indicated the presence of specific differences in *pilA* function between the two *A. citrulli* groups, but the regulatory mechanisms involved require further study.

## 1. Introduction

Bacterial fruit blotch (BFB) is caused by *Acidovorax citrulli* [1] and is one of the most important seed-borne bacterial diseases of cucurbit crops (such as watermelon and melon). The disease causes seedling blight and fruit rot in susceptible cucurbits [2]. Since it was first reported in the United States in 1965, BFB has spread to many countries and regions in the world, causing substantial economic losses in cucurbit production [3,4].

*A. citrulli* shows extensive intraspecies genetic diversity. The species is divided into at least two groups based on clear differences in pathogenicity [5], host preference [6,7,8], and genome sequences [9] between strains. Although both groups of *A. citrulli* strains can infect cucurbitaceae crops such as melon and watermelon, due to the host preferences of different groups of strains, there are significant differences in pathogenicity between the two groups of strains to different hosts [5,6]. Group I strains have strong pathogenicity to both watermelon and melon, while group II strains are more pathogenic to watermelon than melon and less pathogenic to melon than watermelon [7,8]. In the field, group I strains mainly infected the leaves and fruits of non-watermelon cucurbit crops, whereas group II strains mainly infected watermelon leaves and fruits [10,11].

Recent research into the molecular mechanisms associated with *A. citrulli* pathogenesis has largely focused on the type III secretion system (T3SS) [12,13,14,15,16], the type VI secretion system (T6SS) [17,18], the polar flagellum [19], and quorum sensing [20]. Type IV pili (T4P) are known to contribute to *A. citrulli* virulence. These hair-like appendages are found on the surfaces of many bacteria and contribute to key bacterial activities and characteristics, such as surface adhesion, colonization, aggregation, biofilm formation, genetic material uptake, and virulence [21,22].

Genetic and functional characterizations of T4P were first conducted on the mammalian pathogen *Pseudomonas aeruginosa* [23]. The role of T4P in pathogenicity has also been studied in plant pathogens such as *Ralstonia solanacearum* [24], *Xylella fastidiosa* [25], *Xanthomonas* spp. [26], and *Pseudomonas* spp. [27]. In *A. citrulli*, T4P insertion mutants were characterized to determine the genetic function of pili. In the *A. citrulli* group II strain W1, insertion mutants for *pilA* showed reduced virulence, biofilm formation ability, and twitching motility capacity [28]. In another study, 50 mutants impaired in twitching ability were identified among a library of ~1000 transposon mutants in the *A. citrulli* group I strain M6; representative mutants were then further characterized [29].

PilA, one of the core components of the T4P structure and function, is a relatively small subunit (13–23 kDa), thousands of which combine to form T4P. Comparisons of *pilA* gene sequences from plant pathogenic bacteria have revealed extensive genetic variability [30]. A previous study from our lab showed significant sequence diversity in *pilA* between *A. citrulli* strains. Based on differences in the *pilA* sequence, *A*. *citrulli* strains were divided into three types; the sequences of the three types of *pilA* genes can be found in Supplementary Materials Data S1. The sequence identity across the entire gene was 55.81% between types 3 and 1, 56.13% between types 3 and 2, and 61.64% between groups 1 and 2 [31]. We also analyzed the DNA sequences of 31 other T4P genes (Aave_0423, Aave_0532, Aave_0580, Aave_0635, Aave_0637, Aave_0724, Aave_0729, Aave_0732, Aave_0996, Aave_0997, Aave_0998, Aave_0999, Aave_1000, Aave_1422, Aave_2483, Aave_2704, Aave_2716, Aave_2768, Aave_2942, Aave_3156, Aave_3410, Aave_3495, Aave_3549, Aave_3550, Aave_3551, Aave_3552, Aave_3553, Aave_3554, Aave_3680, Aave_3681, Aave_3682) in the *A. citrulli* group II strain AAC00-1 (GenBank accession number NC_008752.1), and the DNA sequences of those genes are identical to that of the group I strain M6 (GenBank accession number NZ_CP029373.1). We therefore hypothesized that differences in the *pilA* DNA sequences may lead to functional differences between the groups.

To test this hypothesis, here, we generated *pilA* deletion mutants, complementation lines, and cross-complementation lines from the group I strain pslb65 and the group II strain Aac5. Pathogenicity, twitching motility, biofilm formation ability, interspecies competition traits, and other phenotypes were assessed in these mutants. Quantitative PCR was also used to analyze the relative expression levels of T3SS-, T6SS-, and T4P-related genes in the two *pilA* deletion mutants to further explore the function of *pilA* in the two groups of *A. citrulli* strains.

## 2. Materials and Methods

### 2.1. Bacterial Strains, Plasmids, and Growth Conditions

The bacterial strains and plasmids used in this study are listed in Table S1. *A. citrulli* strain Aac5 was isolated from watermelon collected in Taiwan, China, and strain pslb65 was isolated from melon collected in Xinjiang, China. *A. citrulli* strains were grown in King’s B (KB) broth or on KB plates at 28 °C. *Escherichia coli* strains were grown in Luria Bertani (LB) medium at 37 °C [32]. Liquid cultures were grown with 200 rpm shaking on a rotary shaker (DDHZ-300; Taicang Experimental Instrument Factory, Jiangsu, Taicang, China). Growth media contained 100 μg/mL ampicillin (Amp), 25 μg/mL chloramphenicol (Cm), 25 μg/mL gentamicin (Gm), and/or 50 μg/mL kanamycin (Km), as specified for each experiment. All antibiotics were purchased from Sigma (Shanghai, China).

### 2.2. Sequence Analysis of pilA in A. citrulli

The DNA sequences of *pilA* and the amino acid sequences of PilA from *A. citrulli* strains pslb65 and Aac5 were aligned using Clustal Omega (https://www.ebi.ac.uk/Tools/msa/clustalo/, accessed on 7 February 2023) and visualized with Jalview v2.11.2.4 [33].

### 2.3. Construction of pilA Gene Deletion Mutants and Complementation Lines

Markerless *pilA* deletion mutants were generated in the *A. citrulli* strains Aac5 and pslb65 using homologous double recombination [34]. The sequences upstream and downstream of *pilA* were amplified from the wild-type (WT) pslb65 and Aac5 strains using the 65AL-F/R and 65AR-F/R primers (for pslb65) and the A5AL-F/R and A5AR-F/R primers (for Aac5) (Table S2). The sequences of the resulting fragments were confirmed via sequencing. The upstream and downstream fragments were concatenated with overlap PCR and then ligated into the pk18mob*sacB* plasmid using the ClonExpress II One Step Cloning Kit (Vazyme Biotech, Nanjing, China) to generate the pK18-pslb65*pilA* and pK18-Aac5*pilA* constructs in *E. coli* DH5α. pK18-pslb65*pilA* and pK18-Aac5*pilA* were then introduced to the *A. citrulli* strains pslb65 and Aac5, respectively, through triparental conjugation using pRK600 as a helper plasmid. Single-exchange colonies were selected on KB plates containing Amp and Km. Individual transformants were continuously sub-cultured in liquid KB medium containing Amp alone and then screened on KB supplemented with Amp and 10% sucrose. The *pilA* deletion mutants for pslb65 and Aac5, pslb65*-*Δ*pilA* and Aac5*-*Δ*pilA*, respectively, were confirmed via PCR using the 65-ΔpilA*-*L/R, A5AL-L/A5AR-R, Km-F/R, and WFB1/2 primers (Table S2). Information on the primer position, product length, and gene knockout position during the construction of *pilA* gene deletion mutants and the PCR validation process can be seen in Figure S1.

To generate complementation lines, the *pilA* genes in pslb65 and Aac5 were amplified using the 65-ΔpilA-L/R and HBA5L/R primers, respectively (Table S2). After confirmation with sequencing, the genes were ligated into pBBR1MCS-2 to create the pBBR-pslb65*pilA* and pBBR-Aac5*pilA* constructs. pBBR-pslb65*pilA* was transferred into the pslb65*-*Δ*pilA* and Aac5*-*Δ*pilA* mutants to generate complementation (pslb65-Δ*pilA*comp1) and cross-complementation (Aac5-Δ*pilA*comp2) lines, respectively, using triparental conjugation. Similarly, pBBR-Aac5*pilA* was transferred into the pslb65*-*Δ*pilA* and Aac5*-*Δ*pilA* mutants to form cross-complementation (pslb65-Δ*pilA*comp2) and complementation (Aac5-Δ*pilA*comp1) lines, respectively. Successful transconjugants were confirmed with PCR.

All primers in this study were designed based on the *A. citrulli* strain AAC00-1 and M6 genomes (GenBank accession numbers NC_008752.1 and NC_CP029373.1, respectively) using Primer Premier v5.0 (PREMIER Biosoft). All primers were synthesized by BGI Laboratories (Shenzhen, China).

### 2.4. Pathogenicity and Hypersensitive Response (HR) Assays

#### 2.4.1. Spray-Inoculation Assays

The virulence levels of each strain (namely, pslb65, pslb65-Δ*pilA*, pslb65-Δ*pilA*comp1, pslb65-Δ*pilA*comp2, Aac5, Aac5-Δ*pilA*, Aac5-Δ*pilA*comp1, and Aac5-Δ*pilA*comp2) were measured in 3-week-old watermelon seedlings (*Citrullus lanatus* cv. ‘Jingxin#3’) and melon seedlings (*Cucumis melo* cv. ‘TVF192’). Briefly, *A. citrulli* strains were cultured overnight in KB broth to an OD_600_ of 0.3 (corresponding to ~3 × 10^8^ colony-forming units (CFU)/mL). The leaves of the melon and watermelon seedlings were spray-inoculated with the bacterial suspension, with sterile water serving as the negative control. Each treatment had 15–20 melon or watermelon seedlings, and 200mL of bacterial suspension was evenly sprayed onto the front and back of the leaves of seedlings in each treatment. At 15 d after inoculation (DAI), the disease severity was determined by measuring the symptoms and calculating the disease index using previously described methods with slight modifications [14]. Seedlings were incubated in a light incubator with a relative humidity of 80% under a 12/12 h light/dark cycle at 26/20 °C.

#### 2.4.2. Stem-Inoculation Experiments

All strains used in spray-inoculation experiments were also used in stem-inoculation experiments, which were performed as previously described [29] with some modifications. Briefly, at 7 d after sowing, the base of the hypocotyl of each seedling (~1 cm above the soil) was inoculated with 10^8^ CFU/mL bacterial suspension using a needle. Seedlings were cultured in a light incubator under the conditions described above for the spray-inoculation assays, and the number of dead seedlings was recorded each day from 3 to 11 DAI. There were 30 seedlings per treatment group (i.e., bacterial genotype) and three independent replicates of the experiment.

#### 2.4.3. HR Assays

To evaluate the effects of *pilA* deletion on HR induction, bacterial cell suspensions (~10^8^ CFU/mL) were injected into the leaves of 3-week-old tobacco (*Nicotiana tabacum* var. *samsun*) plants as previously described [34]. Leaves were visually examined for the HR (tissue collapse) at 24 h after infiltration. Control plants were injected with sterile water. There were three independent replicates of this experiment.

### 2.5. Twitching Motility Assays

Twitching motility was measured in each *A. citrulli* strain using the method described by Bahar et al. [28]. Briefly, *A. citrulli* strains were grown on KB plates containing Amp at 28 °C for 72 h. Each strain was considered to have twitching motility if a thin, light halo was visible around the colonies via light microscopy. An IX83 microscope (Olympus, Japan) was used. Nine plates were inoculated per strain in each replicate experiment, and there were three independent replicates.

### 2.6. Biofilm Formation Assays

Biofilm formation was assessed as previously described [35]. Briefly, 24-well polystyrene cell culture plates (Costar 3524, Corning, NY, USA) were filled with KB broth and then inoculated with a 1:1000 dilution of each *A. citrulli* strain at a concentration of 3 × 10^8^ CFU/mL. The plates were incubated at 28 °C for 48 h without shaking. After incubation, 0.1% crystal violet was added to each well, and the plate was incubated for 30 min at room temperature. Each well was then gently washed three times with distilled water. To quantify biofilm formation, the stained biofilms were solubilized in 95% ethanol for 2 h, and then the OD_575_ value of each stained cell suspension was measured with an Evolution 300 UV/VIS spectrophotometer (Thermo Fisher Scientific, Waltham, MA, USA). There were three biological replicates for each *A. citrulli* strain in each experiment, and there were three independent replicates of this experiment.

### 2.7. Interspecies Bacterial Competition Assays

Interspecies competitions were conducted, with *E. coli* DH5α serving as the prey strain. The killer strains (WT, mutant, and complementation *A. citrulli* strains) were cultured in KB broth to an OD_600_ of 1.5, and the prey culture was grown in LB broth to an OD_600_ of 2.0. The killer and prey cultures were each centrifuged and resuspended in sterile water and then mixed at a 20:1 killer–prey ratio. After co-culture, the mixed cultures were 10-fold serially diluted and spotted on LB plates containing 25 μg/mL Gm. After 3 h, the number of surviving *E. coli* DH5ɑ was quantified in log_10_ (CFU/mL) [18].

### 2.8. Quantitative Reverse Transcription (qRT)-PCR Assays

To assess the effects of *pilA* expression on T3SS genes, the WT *A. citrulli* strains pslb65 and Aac5 and the *pilA* mutants pslb65-Δ*pilA* and Aac5-Δ*pilA* were cultured in the T3SS-inducing broth XVM2 [16]. To assess the effects of *pilA* expression on T4P genes, the same strains were cultured in KB broth. To assess the effects of *pilA* expression on T6SS-related genes, the same strains were also cultured in KB broth to OD_600_ values of 1.0 and 1.5. Total RNA was extracted from each culture using Trizol reagent (Invitrogen, Waltham, MA, USA). cDNA fragments were synthesized using a HiScript II RT SuperMix kit (Vazyme Biotech, Najing, China). *rpoB* was used as the internal reference gene for expression normalization [35]. qRT-PCR was performed on a 7500 Sequence Detection System (ABI, Waltham, MA, USA) with SYBR Green SuperReal PreMix Plus (TIANGEN, Beijing, China) following the manufacturer’s instructions. Relative gene expression was calculated using the 2^−ΔΔCt^ method [36]. The primers used for qRT-PCR are listed in Table S3. There were three technical replicates per sample and three independent replicates of the experiment.

### 2.9. Statistical Analysis

Statistical analyses were conducted using SPSS version 22.0 (SPSS, Chicago, IL, USA) and GraphPad Prism 7.0 software (GraphPad Software, La Jolla, CA, USA). Significant differences between treatment groups were assessed with Student’s *t*-test (for sample pairs) or analysis of variance (ANOVA) and post hoc Duncan’s multiple range test (for multiple samples). Differences were considered statistically significant at *p* < 0.05.

## 3. Results

### 3.1. pilA Sequence Analysis in A. citrulli Strains pslb65 and Aac5

**Figure 1:**
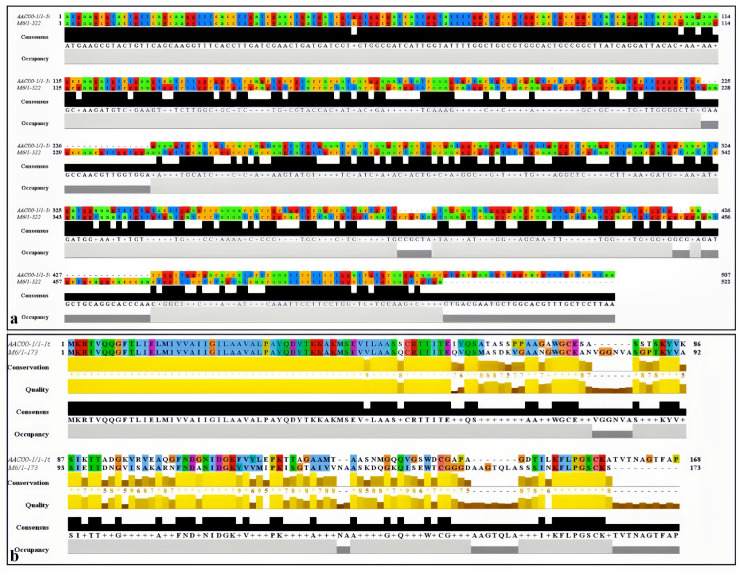
Alignment of *pilA* sequences from *Acidovorax citrulli* strains pslb65 and Aac5. Alignment was performed with Clustal Omega and visualized in Jalview. (**a,b**) Alignment of the (**a**) *pilA* DNA sequences and (**b**) *pilA* amino acid sequences from the two *A. citrulli* strains.

The *pilA* gene of the group I strain pslb65 was 522 bp, and the *pilA* gene of the group II strain Aac5 was 507 bp. DNA sequence analysis revealed that the two *pilA* genes were highly similar from positions 1 to 107 bp, after which the sequences diverged significantly. The overall similarity between the two genes was only 61.64% (Figure 1a). The alignment of the corresponding amino acid sequences showed similar results; amino acids 1–44 were identical, whereas the C-terminal sequences had extensive variation (Figure 1b). SMART (http://smart.embl-heidelberg.de/, accessed on 7 February 2023) did not predict any typical protein domains in either of the two PilA proteins.

### 3.2. Verification of Mutant and Complementation Strains

#### 3.2.1. Knockout Mutant Verification

PCR amplification using the primer pair WFB1/WFB yielded 360 bp amplicons from each of the two putative mutants (pslb65-Δ*pilA* and Aac5-Δ*pilA*), but there was no amplification when the primer pairs Km-F/Km-R, 65-Δ*pilA*-L/65-Δ*pilA*-R, or A5AL-L/A5AR-R were used (Figure S2a,b). The two new lines were therefore confirmed to be *A. citrulli* without the *pilA* gene, indicating successful mutant construction for each strain.

#### 3.2.2. Complementation Line Verification

PCR was performed on the putative complementation lines using the primer pairs WFB1/WFB and Km-F/Km-R. WFB1/WFB2 yielded 360 bp amplicons, and Km-F/Km-R yielded 634 bp amplicons from the four complementation lines (pslb65-Δ*pilA*comp1, pslb65-Δ*pilA*comp2, Aac5-Δ*pilA*comp1, and Aac5-Δ*pilA*comp2) (Figure S2c–f). These results indicated that the four complementation strains were *A. citrulli*, and each contained the pBBR-pslb65*pilA* or pBBR-Aac5*pilA* plasmid. Furthermore, using the primer pair 65-Δ*pilA*-L/65-Δ*pilA*-R, pslb65-Δ*pilA*comp1 and Aac5-Δ*pilA*comp2 yielded identical products, and the primer pair HBA5-L/HBA5-R yielded a separate amplicon that was identical between Aac5-Δ*pilA*comp1 and pslb65-Δ*pilA*comp2. The PCR products were then analyzed via sequencing. Both PCR and sequencing showed that the complementation and cross-complementation lines had been successfully constructed.

### 3.3. The Absence of pilA Did Not Affect the HR in Nonhost Plants

The WT, mutant, and complementation strains of pslb65 and Aac5 were injected into tobacco leaves and cultivated for 24 h. The results showed that ∆*pil*A-pslb65 and ∆*pil*A-Aac5 could cause the HR in a nonhost plant species (Figure S3).

### 3.4. pilA Was Required for A. citrulli Virulence 

To reveal the role of *pilA* in *A. citrulli* pathogenicity, we carried out spray- and stem-inoculation assays.

#### 3.4.1. Spray-Inoculation Assays

**Figure 2:**
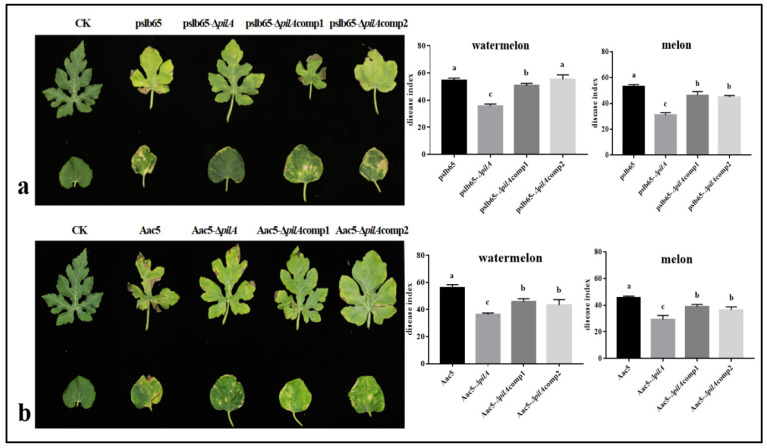
Spray inoculation of watermelon and melon seedlings with wild-type (WT) *Acidovorax citrulli* group I strain pslb65 and group II strain Aac5 and the corresponding knockout mutant and complementation lines. (**a**) Leaves of watermelon (upper) and melon (lower) seedlings inoculated with WT pslb65 and the corresponding knockout mutant and complementation lines. (**b**) Leaves of watermelon (upper) and melon (lower) seedlings inoculated with WT Aac5 and the corresponding knockout mutant and complementation lines. Disease severity was assessed and disease index values were calculated at 15 d after inoculation. Bars show the mean disease index values calculated from three independent replicates. Error bars represent standard error. Lowercase letters above each bar indicate significant differences between strains at *p* < 0.05 (analysis of variance and post hoc Duncan’s multiple range test).

Watermelon and melon seedlings were inoculated with the group I strain pslb65 and the corresponding mutant and complementation strains. Leaf symptoms were observed, and the disease indices were calculated at 15 DAI. The results showed that the average disease index was significantly lower in watermelon inoculated with pslb65-Δ*pilA* compared to WT pslb65. The average disease index was significantly higher in watermelon inoculated with pslb65-Δ*pilA*comp1 compared to the knockout mutant but did not reach WT levels. However, the disease index of watermelon plants inoculated with the pslb65 cross-complementation line (pslb65-Δ*pilA*comp2) was statistically comparable to that inoculated with the WT strain. In melon, the average disease index of seedlings inoculated with pslb65-Δ*pilA* was significantly lower than in those inoculated with WT pslb65. The average disease indices were significantly higher for both pslb65-Δ*pilA*comp1 and pslb65-Δ*pilA*comp2 compared to the knockout mutant but did not return to WT values (Figure 2a).

Watermelon and melon seedlings were also inoculated with the group II strain Aac5 and the corresponding mutant and complementation strains. The average disease index was significantly lower in watermelon inoculated with Aac5-Δ*pilA* compared to those inoculated with WT Aac5. The average disease indices of watermelon treated with Aac5-Δ*pilA*comp1 and Aac5-Δ*pilA*comp2 were significantly higher than those inoculated with the knockout mutant but did not reach WT levels. The average disease index of melon inoculated with Aac5-Δ*pilA* was comparable to the results seen in watermelon (Figure 2b).

#### 3.4.2. Stem-Inoculation Assays

**Figure 3:**
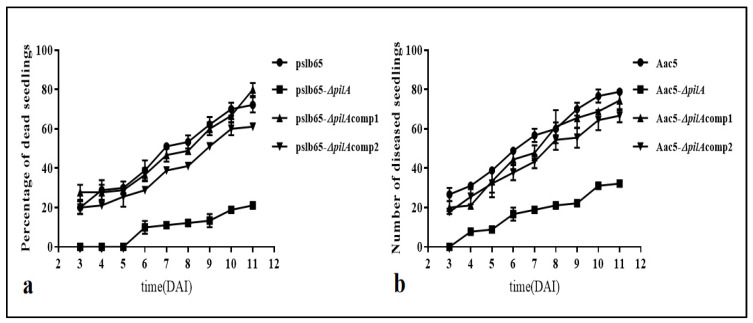
Results of watermelon seedling stem inoculation with *Acidovorax citrulli*. (**a**) Disease rates among watermelon seedlings inoculated with the wild-type (WT) group I strain pslb65 and the corresponding *pilA* knockout mutant and complementation strains. (**b**) Disease rates among watermelon seedlings inoculated with the WT group II strain Aac5 and the corresponding knockout mutant and complementation strains. Seedlings were assessed from 3 to 11 d after inoculation. Dead seedlings were counted as diseased.

In order to further reveal the role of differences in *pilA* between the two groups of strains in *A. citrulli* pathogenicity, watermelon seedlings were inoculated with the group I strain pslb65 and the group II strain Aac5 and the associated knockout mutant and complementation strains. We then measured the number of diseased seedlings from 3 to 11 DAI. The number of diseased seedlings gradually increased over time, and trends were similar between the two groups of strains (Figure 3). At 11 DAI, an average of 21.6 seedlings inoculated with WT pslb65 were diseased (incidence rate = 72.0%). In plants inoculated with pslb65-Δ*pilA*, an average of 6.3 seedlings were diseased (incidence rate = 21.0%). For plants inoculated with the complementation and cross-complementation lines pslb65-Δ*pilA*comp1 and pslb65-Δ*pilA*comp2, the average number of diseased seedlings increased again to 24.0 and 18.3, respectively, representing incidence rates of 80.0% and 61.0%, respectively. Seedlings inoculated with WT Aac5 averaged 23.6 diseased seedlings (incidence rate = 78.6%); this decreased to an average of 9.6 diseased seedlings (incidence rate = 32.0%) among those inoculated with the Aac5-Δ*pilA* mutant. In seedlings inoculated with the complementation line (Aac5-Δ*pilA*comp1), there were an average of 22.3 diseased seedlings (incidence rate = 74.3%), which was comparable to the rate among those inoculated with the cross-complementation line Aac5-Δ*pilA*comp2 (20 seedlings; incidence rate = 66.6%). These results demonstrated that the *pilA* mutants of both pslb65 and Aac5 exhibited low virulence in watermelon seedlings, and that the functional *pilA* genes of group I and group II strains had similar effects on watermelon seedling disease rates.

### 3.5. Twitching Motility Was Impaired in the pilA Mutants of Group I and Group II A. citrulli Strains

**Figure 4:**
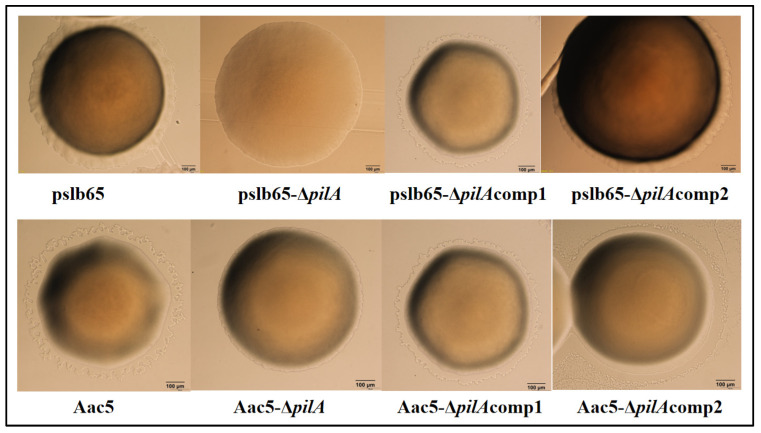
Effects of *pilA* on the capacity for twitching motility in *Acidovorax citrulli.* The group I strain pslb65 and corresponding knockout, complementation, and cross-complementation lines (pslb65-Δ*pilA,* pslb65-Δ*pilA*comp1, and pslb65-Δ*pilA*comp2, respectively) and the group II strain Aac5 and the corresponding knockout, complementation, and cross-complementation lines (Aac5-Δ*pilA,* Aac5-Δ*pilA*comp1, and Aac5-Δ*pilA*comp2, respectively) were grown on King’s B plates with ampicillin at 28 °C for 72 h. Twitching motility was assessed based on the visible presence of a thin, light halo around each colony under light microscopy.

The pslb65-∆*pilA* strain showed no outer halo when it was grown on KB plates, meaning it had completely lost the capacity for twitching motility. In comparison*,* the capacity for twitching motility was significantly reduced in the group II strain Aac5-∆*pilA*, but not completely lost. All four complementation lines (pslb65-Δ*pilA*comp1, pslb65-Δ*pilA*comp2, Aac5-Δ*pilA*comp1, and Aac5-Δ*pilA*comp2) showed the restoration of twitching motility to WT levels (Figure 4).

### 3.6. Biofilm Formation Was Reduced in the pilA Mutants of Strains from Both A. citrulli Groups

**Figure 5:**
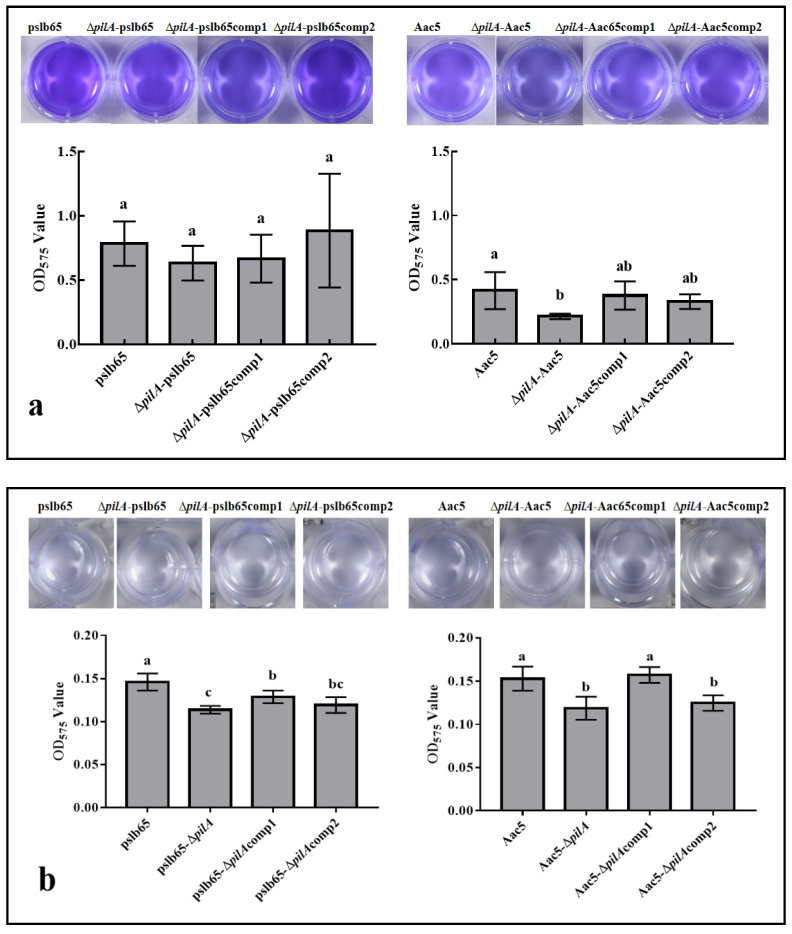
Effects of *pilA* on biofilm formation in King’s B (KB) and M9 broth. (**a**,**b**) Biofilm formation of wild-type (WT) *Acidovorax citrulli* strains pslb65 and Aac5 and corresponding *pilA* mutants and complementation lines grown in (**a**) KB and (**b**) M9 broth. Bars show the mean OD_575_ values from three independent replicates. Error bars represent standard error. Lowercase letters above each bar indicate significant differences between strains at *p* < 0.05 (analysis of variance and post hoc Duncan’s multiple range test).

The effect of the *pilA* gene on the capacity of group I and group II strains to form biofilms was next evaluated in KB and M9 media. In KB medium, pslb65-Δ*pilA* showed decreased biofilm formation compared to the WT strain, but the difference was not statistically significant. However, Aac5-Δ*pilA* showed significantly lower biofilm formation than the WT Aac5 (*p* < 0.05). For both strains, both complementation lines restored biofilm formation to WT levels (Figure 5a). In M9 medium, both pslb65-Δ*pilA* and Aac5-Δ*pilA* showed significantly lower biofilm formation than the corresponding WT strains (*p* < 0.05); all four complementation strains somewhat restored biofilm formation, but only Aac5-Δ*pilA*comp1 reached levels comparable to the WT (Figure 5b).

### 3.7. pilA Was Involved in Interspecies Competition

Interspecific competition is one of the important biological functions of T6SS in bacteria. When the T4P gene *pilA* of *A. citrulli* was deleted, the expression of T6SS genes *hcp* and *vgrG* was affected [18]. To verify the impact of *pilA* gene deletion on the biological function of T6SS, bacterial interspecific competition experiments were conducted. As a weak competitive strain and model strain, *E coli* was used as the prey strain in this test [18].

**Figure 6:**
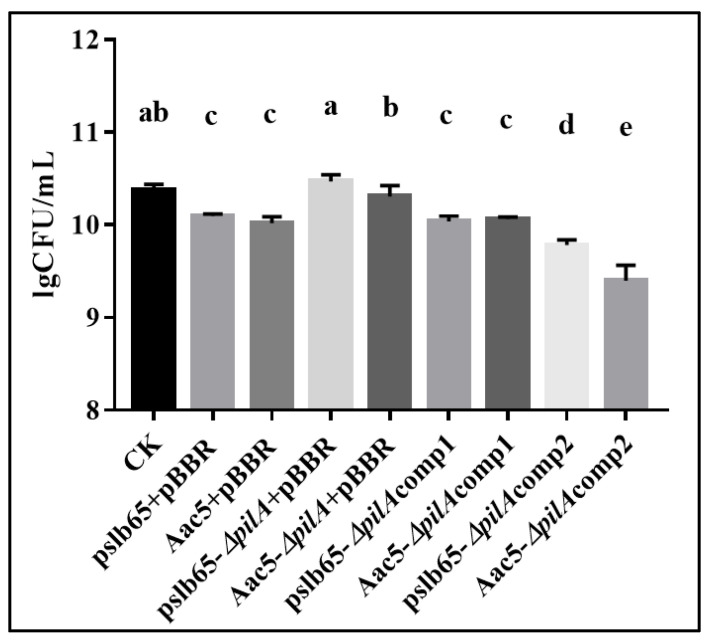
Interspecies competition between *Acidovorax citrulli* and *Escherichia coli* DH5α-pBBRMCS5. Killer cultures (the *A. citrulli* strains pslb65 and Aac5 and the corresponding *pilA* mutants and complementation strains) were combined with the *E. coli* prey culture at a ratio of 20:1. Survival was counted after 3 h of co-culture. Bars show the mean values from three independent replicates. Error bars represent standard error. Lowercase letters above each bar indicate significant differences between strains at *p* < 0.05 (analysis of variance and post hoc Duncan’s multiple range test).

*E. coli* DH5α was co-cultured with each of the eight *A. citrulli* lines assessed in this study, namely, the WT, mutant, complementation, and cross-complementation lines from group I (pslb65, ∆*pilA*-pslb65, pslb65-Δ*pilA*comp1, and pslb65-Δ*pilA*comp2, respectively) and group II (Aac5, ∆*pilA*-Aac5, Aac5-Δ*pilA*comp1, and Aac5-Δ*pilA*comp2, respectively). The *E. coli* and *A. citrulli* colonies were then counted. *E. coli* was significantly more abundant (*p* < 0.05) when co-cultured with pslb65-∆*pilA* compared to WT pslb65. The complementation line (pslb65-Δ*pilA*comp1) completely recovered interspecific competitiveness with *E*. *coli*, whereas colony counts of the cross-complementation line (pslb65-Δ*pilA*comp2) were significantly lower than those of the WT or pslb65-Δ*pilA*comp1 after co-culture with *E. coli*. The results for Aac5 and derivative strains were similar. *E. coli* abundance increased significantly (*p* < 0.05) when it was co-cultured with Aac5-∆*pilA* compared with the WT; Aac5-Δ*pilA*comp1 completely recovered interspecific competitiveness with *E. coli*; and Aac5-Δ*pilA*comp2 competitiveness was significantly decreased compared to both the WT and Aac5-Δ*pilA*comp1 (*p* < 0.05) (Figure 6).

### 3.8. pilA Deletion Affected Expression of Genes Related to T3SS, T4P, and T6SS

#### 3.8.1. Effects of pilA on the Expression of T3SS-Related Genes

**Figure 7:**
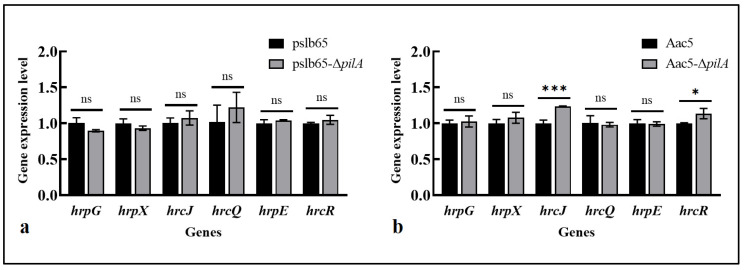
*pilA* deletion affected expression of type III secretion system (T3SS)-related genes in *Acidovorax citrulli*. Expression levels of six T3SS genes were measured via quantitative reverse transcription PCR in the wild-type (WT) strains pslb65 and Aac5 and in the corresponding *pilA* mutants (pslb65-Δ*pilA* and Aac5-Δ*pilA*, respectively). (**a**,**b**) Relative expression levels of the six T3SS genes in (**a**) the group I strain pslb65-Δ*pilA* and (**b**) the group II strain Aac5-Δ*pilA* compared to the corresponding WT strains. Bars show the mean values from three technical replicates per gene in three independent experiments. Error bars represent standard deviations. * *p* < 0.05, *** *p* < 0.001 (Student’s *t*-test). “ns” indicates that there was no significant difference.

The relative expression levels of six T3SS core genes (*hrpG*, *hrpX*, *hrpE*, *hrcJ*, *hrcQ*, and *hrcR*) were measured in pslb65-Δ*pilA* and Aac5-Δ*pilA* to evaluate the effects of *pilA* on T3SS by comparing differences between the two groups of strains. In the group I strain pslb65, *pilA* deletion did not cause significant differences in the expression of six T3SS genes (Figure 7a); for the group II strain Aac5, *hrcJ* and *hrcR* were significantly upregulated in the *pilA* mutant, whereas the other four genes showed no significant differences (Figure 7b).

#### 3.8.2. Effects of pilA on the Expression of T4P-Related Genes

**Figure 8:**
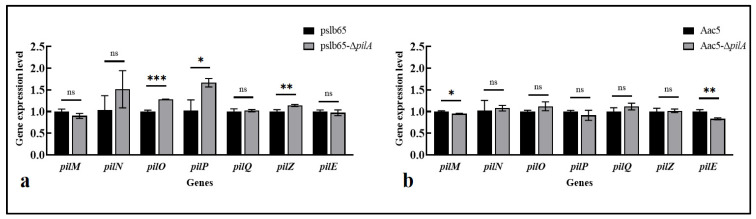
*pilA* deletion affected expression of type IV pili (T4P)-related genes in *Acidovorax citrulli*. Expression levels of 10 T4P genes were measured via quantitative reverse transcription PCR in the wild-type (WT) strains pslb65 and Aac5 and in the corresponding *pilA* knockout mutants (pslb65-Δ*pilA* and Aac5-Δ*pilA*, respectively). (**a**,**b**). Relative expression levels in (**a**) the group I strain pslb65-Δ*pilA* and (**b**) the group II strain Aac5-Δ*pilA* compared to the corresponding WT strains. Bars show the mean values from three technical replicates per gene in three independent experiments. Error bars represent standard deviations. * *p* < 0.05, ** *p* < 0.01, *** *p* < 0.001 (Student’s *t*-test). “ns” indicates that there was no significant difference.

Expression levels of seven T4P-related genes were next analyzed in WT *A. citrulli* and *pilA* knockout mutants. Three genes (*pilO*, *pilP*, *pilZ*) were upregulated to varying degrees in pslb65-Δ*pilA* compared to WT pslb65, while the expression levels of the other four genes showed no significant difference (Figure 8a).

Two genes (*pilM* and *pilE*) were significantly downregulated to varying degrees in Aac5-Δ*pilA* compared to WT Aac5 (*p* < 0.05), and the other five genes showed no significant differences (Figure 8b).

#### 3.8.3. Effects of pilA on the Expression of T6SS-Related Genes

To evaluate the effects of *pilA* deletion on the T6SS in *A. citrulli*, relative expression levels were assessed for 29 T6SS-related genes in pslb65-Δ*pilA* compared to pslb65 and in Aac5-Δ*pilA* compared to Aac5. The tested genes comprised 17 T6SS genes and 12 valine-glycine repeat protein G (VgrG)-encoding genes (*vgrG*s). Because T6SS activation is affected by the concentration of a bacterial suspension, we tested the relative expression of T6SS genes in cultures at an OD_600_ value of 1.5.

**Figure 9:**
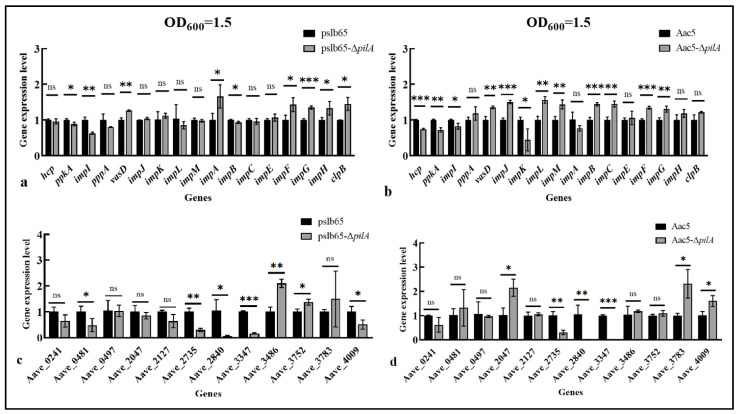
*pilA* deletion affected expression of type VI secretion system (T6SS)-related genes in *Acidovorax citrulli*. A total of 29 T6SS-related genes were selected for study, comprising 17 T6SS genes and 12 *vgrG* genes. Relative expression levels were assessed via quantitative reverse transcription PCR in the wild-type (WT) strains pslb65 and Aac5 compared to the corresponding *pilA* mutants (pslb65-Δ*pilA* and Aac5-Δ*pilA*, respectively). (**a**,**b**) Relative T6SS gene expression levels in pslb65*-*Δ*pilA* (**a**) and Aac5*-*Δ*pilA* (**b**) cultured to OD_600_ values of 1.5. (**c**,**d**) Relative *vgrG* gene expression levels in pslb65*-*Δ*pilA* (**c**) and Aac5*-*Δ*pilA* (**d**) cultured to OD_600_ values of 1.5. Bars show the mean values calculated from three technical replicates per gene in three independent experiments. Error bars represent standard deviations. * *p* < 0.05, ** *p* < 0.01, *** *p* < 0.001 (Student’s *t*-test). “ns” indicates that there was no significant difference.

For pslb65-Δ*pilA*, six T6SS genes (*vasD*, *impA*, *impF*, *impG*, *impH,* and *clp*B) were significantly upregulated; three T6SS genes (*ppkA*, *impI*, and *impB*) were significantly downregulated (*p* < 0.05), and the expression level of *hcp* showed no significant differences compared to the WT strain (Figure 9a). For Aac5-Δ*pilA*, eight genes (*vasD*, *impJ*, *impL*, *impM*, *impB*, *impC*, *impF*, and *impG*) were significantly upregulated; four T6SS genes (*hcp*, *pppA*, *impI*, and *impK*) were significantly downregulated (Figure 9b).

For pslb65-Δ*pilA*, two *vgrG* genes (*Aave_3486* and *Aave_3752*) were significantly upregulated; five genes (*Aave_0481*, *Aave_2735*, *Aave_2840*, *Aave_3347*, and *Aave_4009*) were significantly downregulated (*p* < 0.05) (Figure 9c). For Aac5-Δ*pilA*, three *vgrG* genes (*Aave_2047*, *Aave_3783*, and *Aave_4009*) were significantly upregulated; three genes (*Aave_2735*, *Aave_2840*, and *Aave_3347*) were significantly downregulated (Figure 9d).

## 4. Discussion

Previous studies have found that group I and group II strains of *A. citrulli* exhibit differences in pathogenicity, host preference, and genome sequences [5,8,9]. However, there have been no previous reports of the functional differences in a single gene between the two *A. citrulli* groups. In the present study, the sequence and function of *pilA* were analyzed in the two groups of strains. Significant differences in the sequences between the two groups were found to correspond to differences in biofilm formation ability, twitching motility, and interspecies competition.

Biofilm formation is a process that allows microorganisms to irreversibly adhere to and grow on a surface by producing extracellular polymers that promote adhesion and matrix formation; this enhances the ability of the bacteria to withstand harsh environmental conditions [37]. The results of this study showed inconsistent effects of *pilA* deletion on biofilm formation during growth in KB and M9 media. In M9 medium, *pilA* deletion significantly reduced the capacity for biofilm formation in both strains, consistent with previous reports [28,29]. However, in the nutrient-rich KB medium, the effects of *pilA* deletion differed between strains. In Aac5, the absence of *pilA* significantly reduced biofilm formation, but this was not the case for pslb65. Thus, for the group I strain pslb65, nutrient conditions determined the effects of *pilA* deletion on the capacity for biofilm formation. The relationship between nutrient metabolism and biofilm formation is largely unknown, but it has been suggested that carbon molecules may act as simple nutrients for biofilm-forming bacteria [38]. Alterations in nutrient availability, oxygen depletion, and other environmental factors are also known to affect biofilm dispersion [39]. These factors may be the cause of the observed significant differences in biofilm formation under different nutritional conditions. However, the differences in biofilm formation between *pilA* deletion mutants in the group I and the group II strain under consistent nutrient conditions may have been caused by group-specific differences in the regulatory pathways associated with *pilA*.

Previous research found that the *pilA* and *pilO* mutant strains of *P. syringae* pv *tabaci* 6605 reduced the HR in nonhost *Arabidopsis* leaves, and qPCR results showed that multiple T3SS-related genes of *pilA* and *pilO* deletion mutants were significantly downregulated in expression compared to the WT [27]. However, in this study, we found that there was no significant effect on the HR in nonhost plant tobacco leaves between the *pilA* deletion mutant of *A citrulli* and the WT. The qPCR results showed that the expression of T3SS-related genes in pslb65-Δ*pilA* did not significantly differ compared to the WT, and in Aac5-Δ*pilA* strains, *hrcJ* and *hrcR* were significantly upregulated, whereas the other four genes showed no significant differences. This is different from the research results on *P. syringae* pv *tabaci* 6605. This indicates that in *A citrulli*, the effect of *pilA* on virulence may not be mediated through T3SS, but through alternative pathways. Vfr is a cAMP-binding protein and a transcriptional regulator that can regulate many virulence factors, such as quorum sensing, T4P biogenesis, and T3SS-related genes [40]. Taguchi (2011) believes that there is a global regulatory system between cAMP, Vfr, and T4P that controls bacterial virulence [27]. Through protein sequence alignment, we found that there is a gene homologous to *P. aeruginosa* Vfr in *A. citrulli*. Next, we will further investigate the regulatory relationship between *vfr* and T4P by constructing *vfr* mutants.

The group I strain pslb65 completely lost its capacity for twitching motility when *pilA* was knocked out, consistent with previously published results for the group I strain M6 [29]. Surprisingly, the group II strain Aac5 only partially lost its twitching motility in the *pilA* mutant. To understand the cause of the loss in motility, we identified seven genes that are predicted to have a close regulatory relationship with *pilA* in the group II strain AAC00-1 using the STRING website (http://version10.string-db.org, accessed on 3 May 2022). We then measured the relative expression levels of those seven genes in pslb65-Δ*pilA* and Aac5-Δ*pilA* compared to the corresponding WT strains. Three genes (*pilO*, *pilP*, and pilZ) were upregulated to varying degrees in pslb65-Δ*pilA*, while in Aac5-Δ*pilA,* no genes were upregulated, and two genes (*pilM* and *pilE*) were significantly downregulated to varying degrees compared to WT Aac5. This indicates that there were certain differences in the regulation of T4P by the *pilA* gene between the two groups of *A. citrulli*.

The T6SS plays important roles in pathogenicity, biofilm formation, bacterial interactions, and other biological functions [41]. There is reportedly a T6SS gene cluster in *A. citrulli* that contains 17 genes [17]. Hcp is not only a protein that plays a structural role in the T6SS but also an indispensable special effector in the T6SS [42]. VgrG is a versatile protein that is essential to T6SS function and is involved in bacterial competition. There is some evidence that Hcp and T6SS expression is only activated in the *A. citrulli* strain Aac5 at a cell density of OD_600_ = 1.5 [18]. At that cell density, we found that the interspecific competition experiments with *E. coli* showed no significant differences between the group I strain pslb65 and the group II strain Aac5; however, after *pilA* deletion, the interspecific competition ability of Aac5-Δ*pilA* was significantly higher than that of pslb65-Δ*pilA*, and complementation with endogenous *pilA* returned their competitive abilities to WT levels, whereas cross-complementation significantly enhanced the interspecific competition ability of both strains. To further analyze the effects of *pilA* deletion on T6SS functioning, we analyzed the relative expression of 17 T6SS genes and 12 *vgrG* genes in pslb65-Δ*pilA* and Aac5-Δ*pilA* at cell densities of OD_600_ = 1.5. Interestingly, the experimental results showed that the expression level of the *hcp* gene in Aac5-Δ*pilA* was significantly downregulated (*p* < 0.0001), while there was no significant difference in pslb65-Δ*pilA*. *Aave_3347* (*vgrG* gene) also caught our attention, as its relative expression levels were significantly downregulated (*p* < 0.001) in both pslb65-Δ*pilA* and Aac5-Δ*pilA*. This may be one of the reasons for the decrease in interspecific competition with *E. coli*. The regulatory relationship between the *pilA* gene and *Aave_3347* needs further research.

T6SS activation in *Neisseria cinerea,* a commensal of the human respiratory tract, can limit the growth of related pathogens. Custodio et al. (2020) believe that T4P affects T6SS-mediated antagonism by constructing and distributing bacteria in mixed microcolonies in this process; in other words, spatial segregation driven by type IV pili dictates prey survival against T6SS assault. In the process of bacterial interspecific competition, the *pilA* gene is more sensitive to attack by other bacterial T6SSs and can mount an earlier response [43]. Based on comprehensive analysis, we speculate that the main reason for the decrease in pslb65-Δ*pilA* and Aac5-Δ*pilA* in the interspecific competition of *E. coli* is the loss of spatial isolation caused by the loss of T4P; in addition, key differences in the *pilA*-mediated regulation of T6SS and *vgrG* between pslb65 and Aac5 play a secondary role.

## 5. Conclusions

In the present study, we characterized the function of *pilA* in the *A. citrulli* group I strain pslb65 and in the group II strain Aac5. There were significant differences in phenotype between the two strains when *pilA* was deleted. Specifically, twitching motility was completely lost in pslb65-Δ*pilA*, whereas Aac5-Δ*pilA* only partially lost motility. Biofilm formation was significantly lower for Aac5-Δ*pilA* than for Aac5, but there were no significant differences in biofilm formation between pslb65-Δ*pilA* and the WT pslb65. The deletion of *pilA* also significantly reduced the interspecific competitiveness of pslb65-Δ*pilA* and Aac5-Δ*pilA* compared to the corresponding WT strains. qRT-PCR results showed that significantly more genes related to T4P and the T6SS were upregulated, and significantly fewer were downregulated in Aac5-Δ*pilA* compared with pslb65-Δ*pilA*. These results revealed critical differences in *pilA* function between group I and group II strains, indicating significant intraspecies divergence in T4P regulatory mechanisms.

## Data Availability

Not applicable.

## Supplementary Materials

The following supporting information can be downloaded at https://www.mdpi.com/article/10.3390/microorganisms11071806/s1: Data S1: The sequences of three types of *pilA* genes in *Acidovorax citrulli;* Figure S1: Information on primer position, product length, and gene knockout position during the construction of *pilA* gene deletion mutants and PCR validation process; Figure S2: PCR validation of the mutants and complementary strains; Figure S3: Effects of *pilA* in inducing hypersensitive responses in nonhost tobacco; Table S1: The bacterial strains and plasmids in this study; Table S2: Sequences and related information of primers used for construction of mutant and complementary strains; Table S3: Information about primers used for qPCR in this study. References [44,45,46] are cited in the supplementary materials.
