# Supplementary material for: pilA Gene Contributes to Virulence, Motility, Biofilm Formation, and Interspecific Competition of Bacteria in Acidovorax citrulli
Source: Microorganisms. 2023 Jul 14;11(7):1806. doi: 10.3390/microorganisms11071806 (PMC10385852; doi:10.3390/microorganisms11071806)
Supplement: Supplementary file 1 [file microorganisms-11-01806-s001.zip › microorganisms-2450526-supplementary.pdf]

Supplementary Materials

Data S1: The sequences of three types of *pilA* genes in *Acidovorax citrulli.*

Table S1: The bacterial strains and plasmids in this study;

Table S2: Sequences and related information of primers used for construction of mutant and complementary strains;

Table S3: Information about primers used for qPCR in this study;

Figure S1: Information of primer position, product length, and gene knockout position during the construction of *pilA* gene deletion mutants and PCR validation process.

Figure S2: The *pilA* gene mutant and complementary strains of group I strain pslb65 and group II strain Aac5 were successfully obtained through the verification of gene specific primers of *Acidovorax citrulli*, Kana specific primers and two *pilA* specific primers.

Figure S3: Effects of *pilA* in inducing hypersensitive responses in non-host tobacco

Data S1: The sequences of three types of *pilA* genes in *Acidovorax citrulli.*

The *pilA* gene sequence of type I strain pslb65

The sequence of *pilA* of pslb65 is identical to that of *APS58_1709* of M6 (NZ_CP029373.1)

ATGAAGCGTACTGTTCAGCAAGGTTTCACCTTGATCGAACTGATGATCGTCGTGGCGAT
CATTGGTATTTTGGCTGCCGTGGCACTGCCGGCTTATCAGGATTACACGAAAAAGGCGA
AGATGTCGGAAGTGGTCTTGGCTGCGTCGCAGTGTCGTACCACTATTACCGAGCAGGT
TCAAAGCATGGCATCCGACAAGGTCGGTGCGGCCAATGGTTGGGGCTGTGAAGCCAA
CGTTGGTGGAAATGTTGCATCCGGCCCTACCAAGTATGTTGCCTCTATCGAAACTACTG
ACAACGGCGTGATTTCTGCAAAGGCTCGTAACTTCAACGATGCTAATATCGATGGTAAA
TATGTTGTGATGATCCCTAAAATCTCCGGCACTGCTATCGTGGTCAATGCCGCTAGTAAA
GATCAAGGCAAGCAAATTTCTGAATGGACCTGTGGCGGCGGAGATGCTGCAGGCACC
CAACTGGCTTCCTCTAGTATCAACAAATTCCTTCCTGGCTCCTGCAAGTCGTGA

The *pilA* gene sequence of type II strain Aac5

The sequence of *pilA* of Aac5 is identical to that of *Aave_4679* of AAC00-1 (NC_008752.1)

TTAAGGAGCAAACGTGCCAGCATTCGTCACGGTTGCCTTGCACGATCCAGGAAGGAAT
TTGAGAATGGTGTCGCCAGCCGGTGCACCGCAATCCCAAGAGCCAACTTGCTGCCCCA
TATTGCTAGCAGCAGTCATGGCTGCACCAGCGGTGGTTTTGGGCTCCAAGTACACAAA
CTTTCCATCAATGTTGCCATCATTAAAGCCTTGAGCCTCAACACGCACCTTGCCATCGG
CAGTCGTCTTGATGGATTTCACATACTTACTGGTGGACGATGCACTTTCGCAGCCCCAA
GCACCTGCAGCCGGAGGACTCGAAGCAGTAGCACTTTGATAGATTTCCGTGATGGTGG
TACGGCAGCTGGAAGCCGCCAAGATGACTTCAGACATCTTGGCTTTCTTGGTGTAATCC

TGATAAGCCGGCAGTGCCACGGCAGCCAAAATACCAATGATCGCCACCACGATCATCA
GTTCGATCAAGGTGAAACCTTGCTGAACAGTACGCTTCAT

The *pilA* gene sequence of type III strain tw6

(GenBank assembly accession: GCA_000968215.1, >fig|397945.16.peg.1219)

ATGAAGCGTACTGTTCAGCAAGGTTTCACCTTGATCGAACTGATGATCGTCGTGGCGAT
CATCGGTATTTTGGCCGCCGTGGCACTGCCTGCTTATCAGGATTACACAGTTCGCGCTC
GTGTGTCCGAAGTCATCCTGGCCGCTTCTAGCTGCCGTACGACGGTGACAGATACGGT
GCAAAATGCACCGACTACCAATGTCACATCTGCTTTGACTAATGGTTGCAGCATCACTC
CTACCAAATTCGTTGCTTCCGGCACTACTGATGCGAATGGGGTTATTACCGTGGTTGGC
AACGAAACCACTCTCAAGGGCGATGTGAAGGCAAATGCTAACAGCATCATGCTCAAGC
CTTATGTGGGCACTGCTACTGCACCCACTGCTTTGGATGGTGCCAATGATGGAGGAAA
AACCATTATTGAATGGCGTTGCGGCCCTGCGTCTACTAATCCAATGCCTCCCAAGTACC
TGCCTGGCTCTTGCAAGGCAGCATGA

Table S1 The bacterial strains and plasmids in this study

Strain or plasmid	Description	Reference or source
Strains		
E.coli DH5α	*upE44 ΔlacU169(Φ80lacZ ΔM15) hsdR17 recA1 endA1 gyrA96 thi-1 relA1*	Hanahan, 1983
A.citrulli pslb65	Wild-type group I strain; Amp^r	Yan et al. (2013)
A.citrulli Aac5	Wild-type group II strain; Amp^r	Yan et al. (2013)
Δ*pilA*-Aac5	*pilA* matkerless mutation of Aac5; Amp^r	This study
Δ*pilA*-pslb65	*pilA* matkerless mutation of pslb65; Amp^r	This study
Δ*pilA*-Aac5comp1	Δ*pilA*-Aac5 containing pBBR-Aac5pilA, Amp^r，Km^r	This study
Δ*pilA*-Aac5comp2	Δ*pilA*-Aac5 containing pBBR-pslb65pilA, Amp^r，Km^r	This study
Δ*pilA*-pslb65comp1	Δ*pilA*-pslb65 containing pBBR-pslb65pilA, Amp^r，Km^r	This study
Δ*pilA*-pslb65comp2	Δ*pilA*-pslb65 containing pBBR-Aac5pilA, Amp^r，Km^r	This study
Plasmids		
pK18mobsacB	Suicide vector with *sacB* gene, Km^r	Schäfer et al. (1994)
pK18-Aac5pilA	Suicide vector containing upstream and downstream fragments of *pilA* gene of Aac-5 strain on pK18mobsacB, Km^r	This study
pK18-pslb65pilA	Suicide vector containing upstream and downstream fragments of *pilA* gene of pslb65 strain on pK18mobsacB, Km^r	This study
pBBR1MCS-2	Broad-host range expression vector containing pBluescript II *KS-lacZα*; Km^r	Kovach et al (1995)
pBBR-Aac5pilA	pBBR1MCS-2 containing *pilA* gene of Aac-5 strain with its native promoter; Km^r	This study
pBBR-pslb65pilA	pBBR1MCS-2 containing *pilA* gene of pslb65 strain with its native promote; Km^r	This study

pRK 600	Helper strain in tri-parental mating; Cm^r	This study

Table S2 Sequences and related information of primers used for construction of mutant and complemented strains

Primers	Sequence (5’-3’, restriction enzyme sites are underlined)	Restriction enzyme sites	Product of PCR （bp）
65AL-L	ATCCTCTAGAGTCGACCGTAGGCCCGACCATCAG	*Sal* I	429
65AL-R	GAAGGGGCGAAGGACCAGCATTTCGGCAGTTGGGGC	-	
65AR-L	GCCCCAACTGCCGAAATGCTGGTCCTTCGCCCCTTC	-	541
65AR-R	GGCCAGTGCCAAGCTTAAGATCCGCTTCCTGGGC	*Hin*d III	
65-*△pilA*-L	GCAGGAGCCAGGAAGGAA	-	467
65-*△pilA*-R	TCGTCGTGGCGATCATTG	-	
HB-65*pilA*L	CCCCCTCGAGGTCGACGCAAAATTCGCGTAGGCC	*Sal* I	1220
HB-65*pilA*R	ATTCGATATCAAGCTTGAAGGGGCGAAGGACCAG	*Hin*d III	
A5AL-L	ACATGATTACGAATTCGATGAAATCCCTATCAAACG	*Eco*R I	677
A5AL-R	CTGTTCCTTCGGGAGAGATTTAACCCCCAGCAAGAAAA	-	
A5AR-L	TTTTCTTGCTGGGGGTTAAATCTCTCCCGAAGGAACAG	-	605
A5AR-R	GCCAGTGCCAAGCTTCGGGCGAGAACTACGAGAAG	*Hin*d III	
A5AHBS	CGGTATCGATAAGCTTCTGACGAAATTGTCACTCCC	*Hin*d III	816
A5AHBA	TAGAACTAGTGGATCCACCAACCAAGCCAAACC	*Bam* HI	
HBA5L	GGAGCAAACGTGCCAGCA	-	399
HBA5R	CACCAAGAAAGCCAAGATGT	-	
WFB1	GACCAGCCACACTGGGAC	-	360
WFB2	CTGCCGTACTCCAGCGA	-	
Km-F	TGCTCTGATGCCGCCGTGTT	-	634
Km-R	CGAGGAAGCGGTCAGCCCA	-	

Note: “-” means no restriction enzyme sites.

Table S3 Information about primers used for qPCR in this study

Primers	Sequences（5’-3’）	Product length（bp）
hrpG-F	CTCGCCTGGCTGCTGTT	197
hrpG-L	GCTTGTAGATGTGCTGCTCC	
hrpX-F	GCGCTCACGCAAATGCT	175
hrpX-L	GGCAAGCTCCTCCTGTCCTA	
hrcJ-F	CGGCAAGACCTGGAACG	151
hrcJ-R	GCGACGCCATAGATGAAGC	
hrcQ-F	CGAAATCGCCCTCCACG	165
hrcQ-R	GTGGCCTCGGGTTCGG	
hrpE-F	AACGCATGGTGCTGGCAGAG	110
hrpE-R	GTCAGGATGGACACGCAGGC	
hrcR-F	CCGCAAGTTCCTGGACAAG	153
hrcR-R	GCCGTGAGTTCGGTGAGC	
rpoB-F	GCGACAGCGTGCTCAAAGTG	164
rpoB-R	GCCTTCGTTGGTGCGTTTCT	
Aave-0996-F	ATCAGCGACGGCAACATC	186
Aave-0996-R	CTCGGACTCGACCTGGACC	
Aave-0997-F	GCCGTCGTCTATTGGTGGT	133
Aave-0997-R	GGGCAGCGATTTCCTCTTC	
Aave-0998-F	CTGCTGTGCGTCTTCATCG	192
Aave-0998-R	GTACTGCTGCACCTGCTCG	
Aave-0999-F	CGGCAGCCTGAACAAGAA	87
Aave-0999-R	CCCAGGTAATTGCCCACG	
Aave-1000-F	TTCGCTGAAAACCGCAAT	147
Aave-1000-R	CACCACGAGGTTGTTGCC	
Aave-3410-F	CGGCGTACATCCCGTTCT	96
Aave-3410-R	GCAGCGTGAGCAACACGTA	
Aave-3550-F	CAGTCCATCGTTCAAACCG	113
Aave-3550-R	CTTGAGCACGGAGTCGGTA	
Aave-3682-F	GACTTCCGTGCCGCTCTT	83
Aave-3682-R	GCTTGAACGCCCAATACAC	
Aave-4678-F	AAGCATACTGATTCGGGGC	169
Aave-4678-R	GGCGCTAGCAGACAACCAT	
Aave-4687-F	GAAACCGTCCGCATCTCC	92
Aave-4687-R	AGGAAGGGCTCGATGAGGT	
Aave_1465-F	ATGAAGGTTGAAGGTGCC	99
Aave_1465-R	CGAGTTGGGTTGCGTC	
Aave_1469-F	GCATTGGGGGGATTCG	237
Aave_1469-R	GAACAGAGCAGGAACGCA	
Aave_1471-F	CGGATGGGTGAGCCTG	133
Aave_1471-R	CTCGCGGCAGTAGGTG	
Aave_1473-F	CAGCCACGGCAACAACCAG	269
Aave_1473-R	GCAGCCACGCATCCTCCA	
Aave_1474-F	ATGGCGGGCATCCTCTC	180
Aave_1474-R	GAACAGGCTTTCATTGGG	
Aave_1480R	AGGGCGAGCAGAGCGAC	148
Aave_1480-F	GCCAGGTAGTTGAGGGACA	

Aave_1481R	CGGGCGGCGAAGACT	146
Aave_1481-F	CGTCGTCCATCCCAGC	
Aave_0241-F	ACCGTACTGAAACGGTGGAC	234
Aave_0241-R	CGATGGTCTCGGTCTTGGTC	
Aave_0481-F	ACATCGGCATCGCTGTCCAA	265
Aave_0481-R	TGGAGACGGTGATGTGCTGC	
Aave_0497-F	ATCGGTGAATAACCCCGACG	134
Aave_0497-R	AGGTCTTGAGCGAGTTGAGC	
Aave_2840-F	TGTCACGGGACATCACCAAG	208
Aave_2840-R	ATATTGGTCTGCCCGCCTTC	
Aave_3486-F	AAGAACCGCACGAAGACCAT	221
Aave_3486-R	TTCATGAGCACGTTGGTGGA	
Aave_3783-F	CCGAGGGCATCTACTGGTTC	227
Aave_3783-R	CGGCTTGGTGAAGTCGTAGT	
Aave_4009-F	CAGGACCATCGGCTGTACTC	183
Aave_4009-R	GTAATCCCAGGCACGGTAGG	

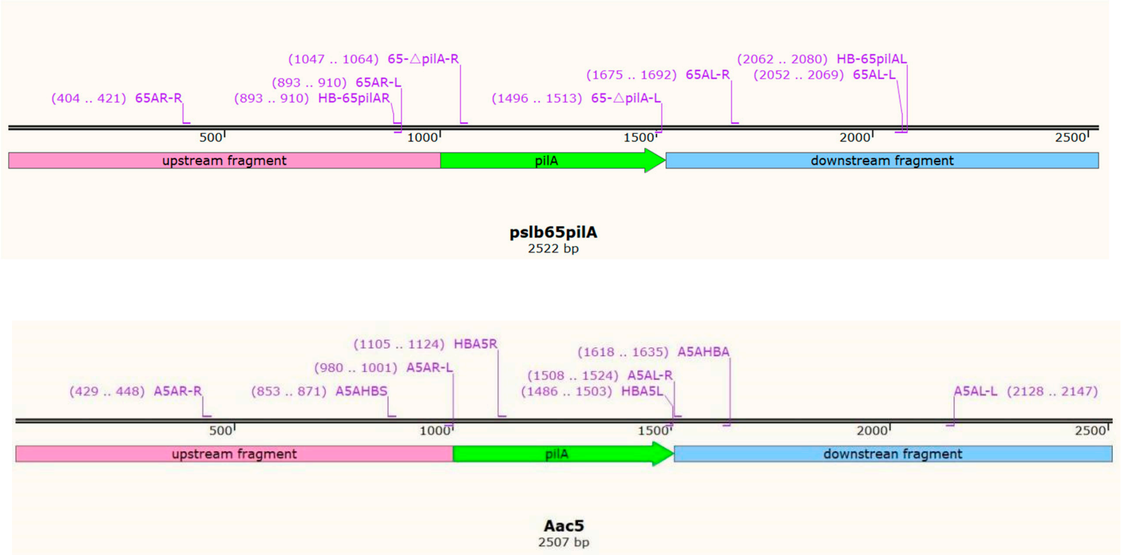

Figure S1. Information of primer position, product length, and gene knockout position during the construction of *pilA* gene deletion mutants and PCR validation process.

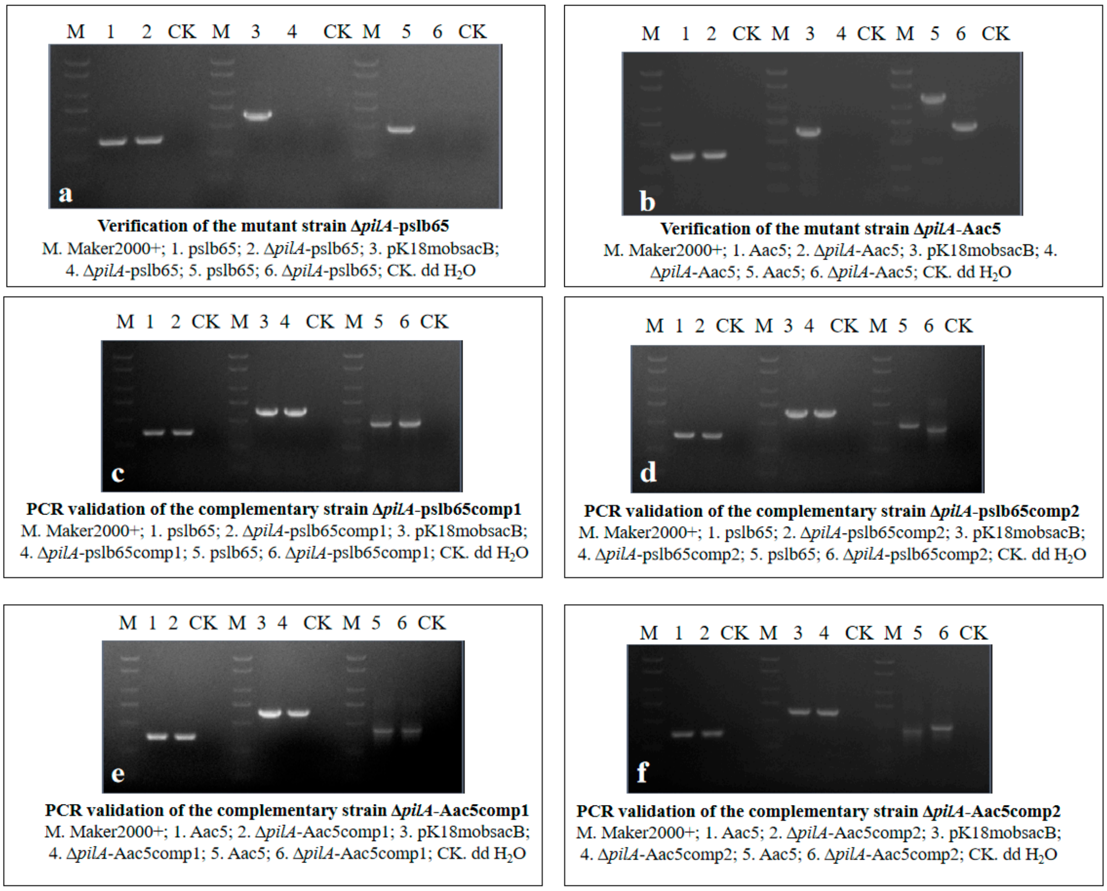

Figure S2 PCR validation of the mutants and complementary strains. The *pilA* gene mutant and complementary strains of group I strain pslb65 and group II strain Aac5 were successfully obtained through the verification of gene specific primers of *Acidovorax citrulli*, kanamycin resistance gene primers and two *pilA* specific primers. a. Verification of mutant strain pslb65-Δ*pilA*; b. Verification of mutant strain Aac5-Δ*pilA*; c. Verification of complementary strain pslb65-Δ*pilA*comp1; d. Verification of complementary strain pslb65-Δ*pilA*comp2; e. Verification of complementary strain Aac5-Δ*pilA*comp1; d. Verification of complementary strain Aac5-Δ*pilA*comp2.

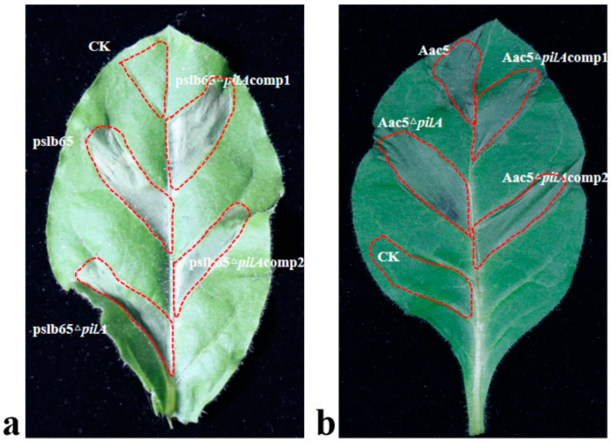

Figure S3. Effects of *pilA* in inducing hypersensitive responses in non-host tobacco. (a) *Nicotiana tabacum* var. s*amsun* leaves inoculated with pslb65，pslb65-Δ*pilA*，pslb65-Δ*pilA*comp1，pslb65-Δ*pilA*comp2 strains, and sterilized water (CK) at 48 h post inoculation. (b) *Nicotiana tabacum* var. s*amsun* leaves inoculated with Aac5，Aac5-Δ*pilA*，Aac5-Δ*pilA*comp1，Aac5-Δ*pilA*comp2 strains, and sterilized water (CK) at 24 h post inoculation.
